# Lateral tongue bite in patient with transient loss of consciousness

**DOI:** 10.1002/ccr3.5264

**Published:** 2022-01-08

**Authors:** Kaho Onizawa, Taku Harada, Juichi Hiroshige

**Affiliations:** ^1^ Center of Postgraduate Clinical Training Showa University Koto Toyosu Hospital Tokyo Japan; ^2^ Division of General Medicine Showa University Koto Toyosu Hospital Tokyo Japan; ^3^ Division of Diagnostic and Generalist Medicine Dokkyo Medical University Hospital Tochigi Japan

**Keywords:** seizure, tongue bite, transient loss of consciousness

## Abstract

The finding of a tongue bite is infrequent since it is a physical finding that is often overlooked, but it has a very high diagnostic value. It is important to check for tongue bites when examining any patient with a transient loss of consciousness.

## Graphical abstract

Tongue bite on the right side due to epilepsy.

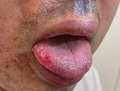

## 1 INTRODUCTION

**FIGURE 1:**
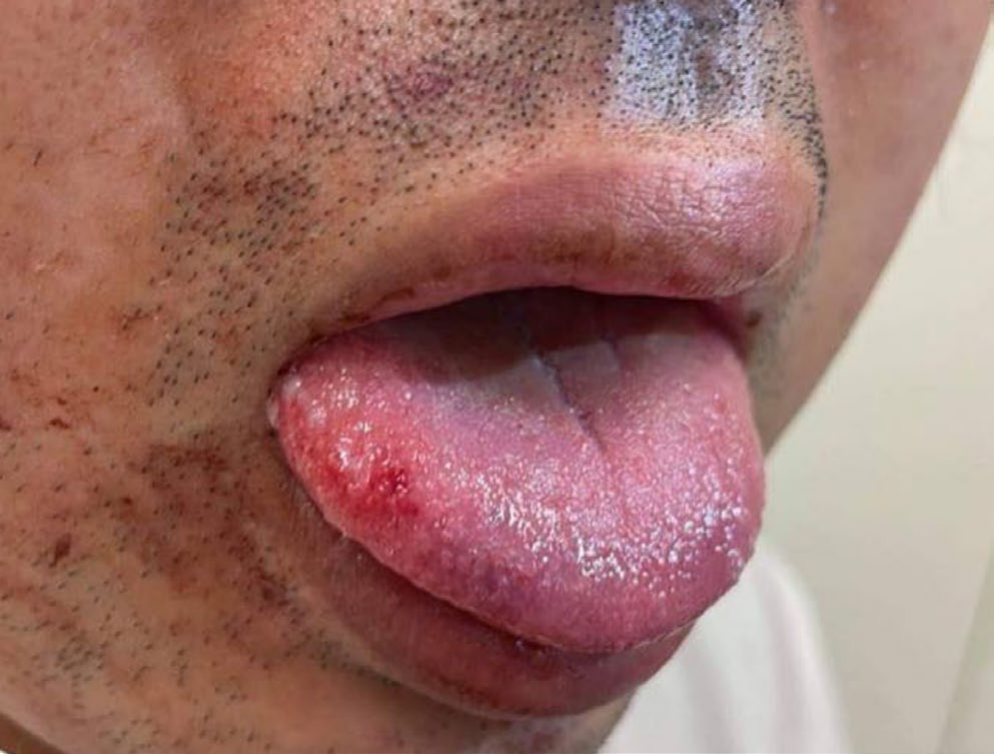
Tongue bite on the right side

A 28‐year‐old man was brought to the emergency room with transient loss of consciousness (TLoC). He experienced temporary memory loss after a fall of unknown cause. At 18, he was diagnosed with epilepsy and was treated with valproic acid but stopped medications for three months. A physical examination revealed no cardiac or neurological abnormalities, and the orthostatic hypotension test was negative, but a lateral tongue bite wound was found (Figure 1). Epilepsy recurrence was diagnosed, and the patient was referred to the outpatient clinic and resumed valproic acid.

More than 90% of TLoC cases are caused by epileptic seizures, syncope, or psychogenic non‐epileptic seizures. Presence of a tongue bite has 33% sensitivity, 96% specificity, and an 8.167 positive likelihood ratio for differentiating epilepsy from syncope.^1^ Furthermore, tongue bites on the lateral side are more specific to epilepsy compared with anterior‐side bites.^2^

For TLoC cases, obtaining information from witnesses is difficult, making the diagnosis challenging. A tongue bite finding is infrequent since it is physical and often overlooked because of the fixation on trauma, neurological, or cardiovascular assessments but has high diagnostic value. It is important to check for tongue bites when examining patients with TLoC.

## CONFLICT OF INTEREST

We have no potential conflicts of interest related to this manuscript.

## AUTHOR CONTRIBUTIONS

According to the definition given by the International Committee of Medical Journal Editors (ICMJE), the following individuals qualify for authorship based on their substantial contributions to the manuscript's intellectual content: Kaho Onizawa and Taku Harada, conception and design and patient management and interpretation of data; Kaho Onizawa, acquisition of data;. Furthermore, Kaho Onizawa and Taku Harada have participated in writing the manuscript. All authors have read and approved the manuscript.

## CONSENT

During submission, it was confirmed that patient consent has been signed and collected in accordance with the journal's patient consent policy.

## Data Availability

Data sharing not applicable to this article as no datasets were generated or analysed during the current study
